# Selectivity of MOFs and Silica Nanoparticles in CO_2_ Capture from Flue Gases

**DOI:** 10.3390/nano13192637

**Published:** 2023-09-25

**Authors:** Felicia Bucura, Stefan-Ionut Spiridon, Roxana Elena Ionete, Florian Marin, Anca Maria Zaharioiu, Adrian Armeanu, Silviu-Laurentiu Badea, Oana Romina Botoran, Eusebiu Ilarian Ionete, Violeta-Carolina Niculescu, Marius Constantinescu

**Affiliations:** National Research and Development Institute for Cryogenic and Isotopic Technologies—ICSI Ramnicu Valcea, 4 Uzinei Street, P.O. Box Raureni 7, 240050 Ramnicu Valcea, Romania

**Keywords:** adsorption, carbon dioxide, column-reactor system, nanomaterials

## Abstract

Until reaching climate neutrality by attaining the EU 2050 level, the current levels of CO_2_ must be mitigated through the research and development of resilient technologies. This research explored potential approaches to lower CO_2_ emissions resulting from combustion fossil fuels in power plant furnaces. Different nanomaterials (MOFs versus silica nanoparticles) were used in this context to compare their effectiveness to mitigate GHG emissions. Porous materials known as metal–organic frameworks (MOFs) are frequently employed in sustainable CO_2_ management for selective adsorption and separation. Understanding the underlying mechanism is difficult due to their textural characteristics, the presence of functional groups and the variation in technological parameters (temperature and pressure) during CO_2_-selective adsorption. A silica-based nanomaterial was also employed in comparison. To systematically map CO_2_ adsorption as a function of the textural and compositional features of the nanomaterials and the process parameters set to a column-reactor system (CRS), 160 data points were collected for the current investigation. Different scenarios, as a function of P (bar) or as a function of T (K), were designed based on assumptions, 1 and 5 vs. 1–10 (bar) and 313.15 and 373.15 vs. 313.15–423.15 (K), where the regression analyses through Pearson coefficients of 0.92–0.95, coefficients of determination of 0.87–0.90 and *p*-values < 0.05, on predictive and on-site laboratory data, confirmed the performances of the CRS.

## 1. Introduction

Earth’s climate has continuously undergone changes; however, the current rate of global warming is unprecedented, particularly within the past 22,000 years [1]. This unparalleled transformation has raised concerns that some components of the climate may exceed a critical threshold [2,3,4]. It is widely accepted that the Earth’s climate system has experienced substantial and abrupt changes in the past when it exceeded certain thresholds [5,6]. Furthermore, even if atmospheric greenhouse gas (GHG) levels are reduced, the Earth’s climate system may not permanently revert to its initial state within a given period, indicating the presence of irreversibility and hysteresis within the climate system [7]. To address the limitations of the liquid amine process stream, one potential solution lies in the utilization of solid adsorbents. In general, solids are easier to handle and pose fewer corrosion problems. Absorption can be split into physical, which is dependent on temperature and pressure, and chemical, where CO_2_ absorption depends on the acid–base neutralization reaction. Adjusting the properties of adsorbents is a solution for activating the selective separation capacity, diffusion selectivity and adsorption properties. The capture of CO_2_ by physical adsorption, adsorbed by the substrate, is much more efficient from an energetic point of view compared to chemical absorbents, due to the absence of the formation of new chemical bonds between the sorbate and the sorbent, small binding energies and large bonding distances of CO_2_ to the surface, thus requiring much less energy for regeneration. Inside the adsorbent, attractive forces act on the adsorbed molecule that balance each other; on the other hand, on the surface, the attractive forces are unbalanced or residual [8]. The robust geometry and crystallography of MOFs allow removal of the included guest species, resulting in permanent porosity. Also, MOFs possess dynamic, “soft” frameworks that respond to external stimuli, such as pressure, temperature and injected molecules, which leads to a molecular sieving mechanism. The physics of gas–sorbent interactions at the molecular level, with respect to the design of the molecular cavern for CO_2_, represent the key element to be researched [9]. The specialized literature reports a wide range of adsorption capacity values (*a* cm^3^/g), as a result of some tests occurring under physisorption mechanisms, with values between 0.784 and 156.8 cm^3^/g [10]. These are explained by the structural differences between the materials (*MOFs*, *nanoparticles*, etc.) chosen for the experiments, specific surface area (100–2000 m^2^g^−1^) and pore volume (0.1–0.5 cm^3^/g^−1^), but also the technical conditions specific to the tests: T (K), P (bar), Q (ml/min) and reactor types (design, capacity, etc.) [10,11]. The fixed-bed reactor is the most common type of cylindrical support plant filled with catalysts and inert ceramic balls to support the catalyst and improve the flow and distribution of the process gas or liquid. The reactor can have several configurations, including one large bed or multiple beds in series [12]. A tubular reactor/reformer consists of a series of tubes containing the catalyst and is normally operated at high temperatures. The processes are highly exothermic, necessitating the placement of the coolant on the exterior of the tube. The use of a single bed/layer in such a reactor configuration presents challenges in obtaining and maintaining desired temperatures [13]. In contrast, a fluidized bed reactor consists of multiple vessel units that suspend small particles of catalyst through the upward movement of the fluid with which reaction occurs. The fluid is usually a gas with a flow rate high enough to mix the particles without removing them from the reactor [14]. The phenomenon of climate change is an ongoing process, and the devastating effects are already present in every region on Earth in multiple ways, including floods, huge fires of vegetation and long droughts, the extent and frequency of which have intensified in the last decade, which causes biodiversity loss and threatens human health. Consequently, researchers from different fields of activity, energy, agriculture and waste have tried to develop beneficial practices to mitigate the effects of climate change, and the present study is no exception.

In this context, our study aims to yield immediate and quantifiable environmental benefits, such as preserving biodiversity and enhancing overall quality of life. As part of this endeavour, a column-reactor system filled with dedicated MOFs (metal–organic frameworks) and silica-derived nanomaterials, able to manage flue gases generated by a conventional power plant, was designed and tested.

MOFs represent crystalline materials with a periodic one-, two-, or three-dimensional coordination matrix designed by metal-based nodes covalently bonded to bridging organic ligands [15]. A significant number of investigations have already been conducted, mainly on CO_2_ capture and conversion [15]. The two synthesised MOFs (Fe-BTC and UiO-66) were chosen due to their proven adsorption capacities [16,17]. By comparison, silica-derived nanomaterials are well-known as CO_2_ adsorbents with high adsorption capacity and selectivity [18]. Fe-BTC, a MOF that has Fe(III) as the metallic centre and trimesic acid (BTC = 1,3,5-benzenetricarboxylate) as the organic linker, was recognized as a valuable material for selective adsorption processes [19]. Also, UiO-66, a Zr-based MOF with terephthalic acid as organic linker, has potential as adsorbent due to its large surface area, easily tuneable pore matrix and high stability [17]. The CO_2_ adsorption properties of mesoporous materials can be improved by introducing transition metals (copper, nickel, chromium, iron, or cerium) and require low regeneration energies [20]. However, there are few studies on the adsorption behaviour or adsorption mechanism of CO_2_ on these transition metal oxides. It is therefore of interest in this study.

The major objective of the present study was to investigate potential solutions to reduce CO_2_ emissions resulting from the combustion of fossil fuels in the boilers of power plants. Various nanomaterials (MOFs versus silica nanomaterials) were applied in order to compare their efficiency in emissions reduction.

## 2. Materials and Methods

### 2.1. Nanomaterials Preparation

All reagents were purchased from Sigma-Aldrich Chemie GmbH (Taufkirchen, Germany) and used without further processing.

#### 2.1.1. Fe-BTC Synthesis

A previous method [19], adapted and modified, was used to prepare the Fe-BTC. In a typical synthesis, 9.2 nmol of anhydrous FeCl_3_ was mixed with 5.4 mmol of 1,3,5-benzentricarboxylic acid (H_3_BTC) in 40 mL of ultrapure water. The resulting suspension was stirred in a magnetic bath at room temperature, leading to the immediate formation of a light orange precipitate. The Fe-BTC formation reaction was monitored for 6 h for the formation of Fe-BTC crystals. Then, the resulting product was centrifuged at 4500 rpm for 10 min in 15 mL tubes. The precipitate was washed with ultrapure water and ethanol and dried under vacuum at 373 K.

#### 2.1.2. UIO-66 Synthesis

A modified old method [21] was used to synthesize the UiO-66. First, 25 mmol of ZrCl_4_ was weighed into a 250 mL Erlenmeyer beaker and then 25 mmol of terephthalic acid was added. Amounts of 45 mL of acetic acid, 2.7 mL of ultrapure water and 150 mL of DMF were then added to the mixture and stirred on a magnetic plate at 303–313 K for 4 h, until the solids were almost completely dissolved. Then, the mixture was divided into two Teflon vessels and subjected to hydrothermal synthesis in a closed enclosure at 393 K for 24 h. After synthesis, Teflon dishes were cooled in ice water for 30 min, after which the mixture was centrifuged at 6000 rpm for 10 min. The supernatant was removed and the precipitate was washed with DMF, followed by 20 mL of acetone, and then dried under vacuum at 323 K.

#### 2.1.3. Cu–Zn–MCM-41 Synthesis

The support material (MCM-41) was prepared by adapting a previously developed method [22]. Briefly, 7.7 mmol of cetyltrimethylammonium bromide (CTAB) was dispersed in 30 g of ultrapure water and the mixture was stirred for 2 h at ambient temperature. Next, 31.1 mmol of sodium silicate was added dropwise under continuous stirring for 2 h. After this time, 240 mmol of tetramethylammonium hydroxide (TMAOH) was added and the mixture was stirred for another 30 min. The pH was adjusted with concentrated sulfuric acid to 10.5. After 10–15 min, the pH was checked and the mixture was left under stirring overnight, after which it was placed in a Teflon autoclave at 373 K for 5 days. The mixture was filtered under vacuum, washed with ultrapure water and dried. The resulting white powder was calcined at 823 K for 6 h to remove excess surfactant. The support, MCM-41 (1 g), was impregnated with the aqueous solution of Cu nitrate—Cu(NO_3_)2.2.5 H_2_O (0.22 g). After impregnation, the precursor was dried for 24 h, alternately in air and at 353 K under vacuum, and then calcined up to 823 K for 6 h. To obtain the bimetallic material, the precursor containing Cu was re-impregnated with Zn nitrate—Zn(NO_3_)2.6 H_2_O (0.0455 g). The previous drying and calcination procedure was repeated, resulting in a light grey product.

### 2.2. Nanomaterials Characterisation

The specific surface area and pore distribution were investigated using a Quantachrome Autosorb-IQ porosity equipment (Quantachrome Instruments, Boynton Beach, FL, USA) by Brunauer–Emmett–Teller (BET) and Barrett–Joyner–Halenda (BJH) methods. Prior to analysis, the nanomaterials were degassed at 373–393 K. The morphology of all samples (before and after adsorption) was investigated using a FESEM VP Scanning Electron Microscope (Carl Zeiss, Oberkochen, Germany) at 0.8 nm resolution, 2.5 nm VP mode and 30 kV; also, EDS was applied for the determination of metal content. Functional groups were elucidated using a Cary 630 ATR-FTIR spectrophotometer (Agilent Technologies, Inc., Santa Clara, CA, USA). Prior to analysis, the samples were ground in an agate mortar and dried at 80 °C under vacuum. The FTIR spectra were registered between 4000 and 400 cm^−1^ (32 scans at a resolution of 8 cm^−1^ and a threshold of 0.002), and MicroLab Expert v.1.0.0 Software (Agilent Technologies, Inc., Santa Clara, CA, USA) was used for the interpretation.

The elemental analysis was achieved by combustion and pyrolysis coupled with gas chromatography (Flash EA2000, Thermo Scientific, Waltham, MA, USA) [23].

### 2.3. Adsorption Tests

To achieve the major objective, sampling campaigns of flue gases were conducted over several months, as indicated in Table 1. The sampling was carried out during periods of natural gas combustion and coal combustion.

**Figure 1:**
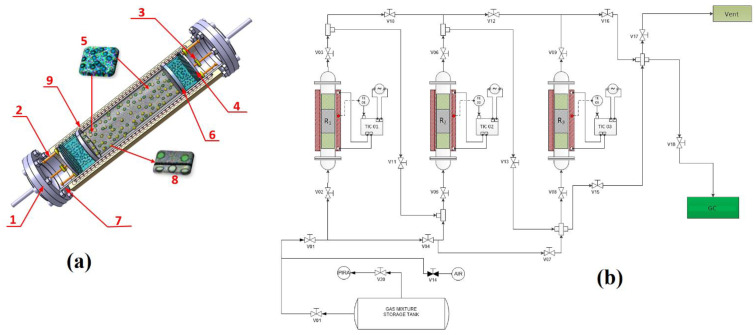
The 3D image of the adsorption column-reactor system (**a**) and the system flowsheet (**b**). The prototype installation for testing selected catalysts presents the following technological components: (**a**) column-reactors, 3 in number, made of stainless steel material: (1) flange system; (2) threaded bar system/centerer; (3) threaded welded clamping lugs; (4) pressing ring with movable cutout-welded mesh; (5) chamber for secondary filling, mix of quartz balls and quartz wool; (6) fixed ring without welded mesh; (7) pipe body (306 L); (8) filler space, nanomaterial mix and quartz balls; (9) variable-controllable thermostatic ceramic heating coil. Specific parameters of the column-reactor: (i) total height H_t_ = 274 mm; (ii) useful height (between the column flanges) H_u_ = 260 mm; (iii) outer diameter Ø = 54 mm; (iv) catalytic filling space height H_c_ = 135 mm. A column-reactor is mounted in a vertical position and loaded with the selected MOFs. (**b**) The AMC equipment (RED-BOX TCS, USA) allows the control of the installation for the operating parameters, respectively, for the thermostatic collars of the column-reactor system, which have the following technical characteristics: (i) inner Ø = 54 mm; (ii) power/necklace: 500–700 W; (iii) thermostatic collar area on column-reactor: 120 mm; (iv) wires covered in metallic braid; (v) connection with protective box; (vi) thermocouple, range of 288–623 K; (vii) type of screw tightening. AMC equipment is provided with connection blocks/junction boxes and connectors resistant to water, dust and possible chemical contaminants. The flue gases from the exhaust stacks of the power plants, as well as the binary test mixture and the gas resulting from the reactions with selected materials, were investigated both qualitatively and quantitatively to determine the evolution of CO_2_ concentrations by the gas-chromatographic method (GC CP3800 Varian Inc., Palo Alto, CA, USA) [24,25]. The binary test mixture of CO_2_ (15.89 vol%) and N_2_ (as balance) was carried out using the partial pressure method [26]. Combustion method coupled with gas chromatography (Flash EA2000, Thermo Scientific, Waltham, MA, USA) was used under the conditions of a reactor filled with CuO, electrolytic copper and quartz wool, with T_reactor_ = 677 K and m_nanomaterial_ = 0.3 ± 0.05 mg, and the amino acid BBOT for the calibration curve, to perform the evolution of the elemental carbon (C wt.%) content in the selected materials [27].

Performance testing of the selected nanomaterials, such as Fe-BTC, UIO-66 or Cu–Zn–MCM-41, was conducted using an experimental column-reactor system of our own design (Figure 1). 

The results of the analysis were obtained through multiple attempts with an RSD < 5%. In addition to instrumental investigations using GC, EA and FTIR, the performance evaluation of the column-reactor system included

(1) $$a=\frac{Q\times p\left(c_{i}-c_{f}\right)\times t}{m}$$

where a = adsorption capacity, cm^3^/g; Q = the flow of the test gas passed over the adsorbent materials, cm^3^/s; p =adsorption pressure, bar; c_i_ = CO_2_ concentration in the cylinder (feed gas), vol%; c_f_ = CO_2_ exit concentration up to adsorbent saturation, vol%; t = breaking time, s and m = the amount of the adsorbent materials in the reactor, g [28];

(2) $$\eta =\frac{c_{a}}{c_{i}}\times 100$$

where η = separation efficiency, %; c_a_ = adsorbed CO_2_ concentration, vol% and c_i_ = initial CO_2_ concentration, vol% [20] and

(3) $$R_{{CO}_{2}}=\frac{{\bar{C}}_{e({CO}_{2})}-C_{i({CO}_{2})}}{C_{i({CO}_{2})}}\times 100$$

where R = degree of recovery, vol%; ${\bar{C}}_{e}$(CO_2_) = the average of the CO_2_ concentrations until the breaking period at the exit from the adsorbent materials, vol% and C_i_ (CO_2_) = initial CO_2_ concentration, vol% [29].

## 3. Results

### 3.1. Characterisation and Behaviour of the Fe-BTC

Due to Fe’s low toxicity and excellent biocompatibility, expanded usage of Fe-BTC in the GHG adsorption niche is typically viewed as an exceptionally desirable goal [30]. The tests conducted, along with related validations using GC, EA and FTIR, revealed a MOF, Fe-BTC, which has selective adsorption capacity—affinity for the investigated gas mixture—as a result of a molecular sieving effect [10]. Technically, the process involved molecules with the appropriate kinetic diameter, which were blocked and trapped in the pores of the Fe-BTC structure (CO_2_—3.3 Å). Also, the tests performed demonstrated that, even during the synthesis of Fe-BTC, the parameters were optimized (in terms of BET surface area and total volume of the pores) to increase the amount of adsorbed CO_2_; ultramicropores and narrowed micropores had a critical role for the adsorbed CO_2_, as presented in Table 2. The adsorption capacity, *a* = 188.11 cm^3^/g, was superior when Fe-BTC was used, by 27.5 wt.% (around 140 cm^3^/g at P = 8.5 bar and T = 298 K [31]), which can be correlated with the average of other BTC-type MOFs (188-210 cm^3^/g) [32,33], at T = 273.15–293.15 K and P = 1 bar.

The physical phenomenon of adsorption starts with the occupation of side rings, followed by the filling of unoccupied positions—channels around the unsaturated Fe atoms filled with CO_2_ at a pressure that is slightly increased in relation to that set in the gas regulator [34]. The presence of micropores (<0.7 nm) has been reported to be crucial for high CO_2_ adsorption at ambient pressures [35]. The loading of Fe-BTC with CO_2_ is facilitated by the electrostatic interaction of the molecule with the open or exposed metal sites of the MOF [36]. Also, CO_2_ molecules could form coordination compounds on Lewis sites in mesoporous MOFs, further enhancing CO_2_ adsorption and creating the premises for CO_2_ solubility inside this structure [37]. In addition, the Fe-BTC particles facilitate gas diffusion, providing an extra porous network for the transport of CO_2_ molecules. To validate the adsorption behaviour of Fe-BTC, a representative and defining investigation was carried out.

**Figure 2:**
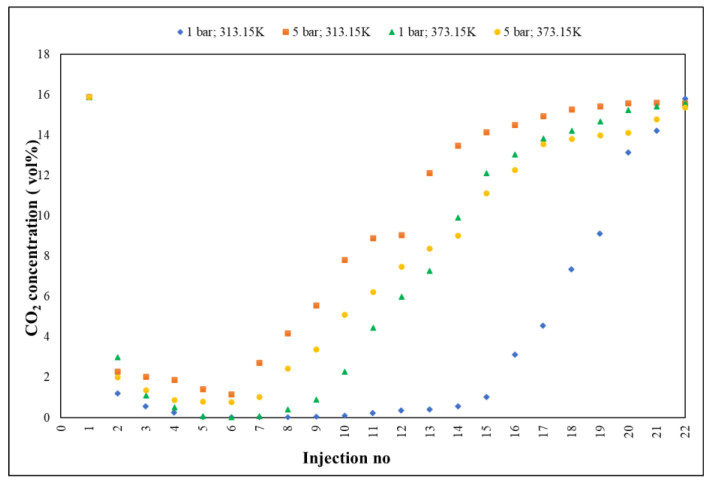
Fe-BTC evolution of selective CO_2_ adsorption by GC investigation.

Gas chromatography (Figure 2) was performed to qualitatively and quantitatively determine the adsorption.

The method followed, in real time, the accelerated slope of decrease of CO_2_ concentration,1(5.89 vol%, reference material/initial value in house) towards 0.02 vol% (~200 ppm ± 5%), the lowest value recorded by GC, with RSD < 3%, followed by a slow return (deceleration) towards the saturation process of Fe-BTC (15.79 vol% ± 5%), the maximum measured value. Practically, the adsorption process was defined by three distinct segments: the first, ranging from 15.89 to 0.02 vol%, displaying a decrease rate of 2.24 vol%; the second, 0.02–0.55 vol%, characterized by an adsorption level, with a rate of 0.05 vol% and the third, 0.55–15.79 vol%, representing the saturation period, with a growth rate of 1.35 vol%.

Elemental analysis (EA), namely, the qualitative and quantitative determination of C (wt.%) from the Fe-BTC sample, was performed using a hybrid investigation method: combustion coupled with gas chromatography [38]. The objective of this investigation was to examine the evolution of CO_2_ as C in the Fe-BTC sample and the behaviour of the samples collected before and after the experiments from the developed MOF column-reactor system, with P = 1–5 bar; T = 313.15–373.115 K and Q: 100 mL/min as working conditions.

The results of the EA investigation revealed an increase in the C concentration at the end of the experiment compared to the initial levels, from C_initial_ = 34.68 wt.% up to C_final_ = 39.62 wt.%.

The EA results, in conjunction with the findings from the GC and FTIR investigation, validated the high affinity for the selective adsorption of Fe-BTC towards CO_2_ from a gaseous mixture.

**Figure 3:**
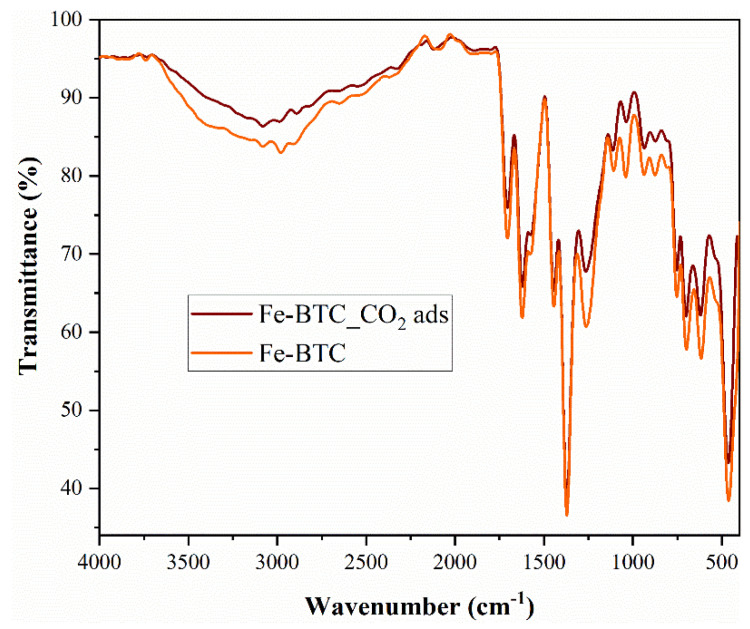
FTIR spectra for Fe-BTC before and after adsorption.

FTIR analysis (Figure 3) confirmed the presence of CO_2_ molecules on the surface of the nanomaterial. The intense bands at 1371 and 752 cm^−1^ confirmed the presence of the trimesic linker in Fe-BTC [39]. Therefore, the Fe-BTC material not only has a similar topology to a commercial MOF, named Basolite F300, but also exhibits a similar bonding environment. This suggests that the nature of the synthesized material, Fe-BTC, is probably equivalent to Basolite F300. After CO_2_ adsorption, the intensity of Fe-BTC specific peaks and bands decreased following adsorption (Figure 3). The lack of CO_2_ molecule- specific peaks does not imply that CO_2_ is not present, but can be correlated with the fact that it is physically adsorbed on the nanomaterial surface.

**Figure 4:**
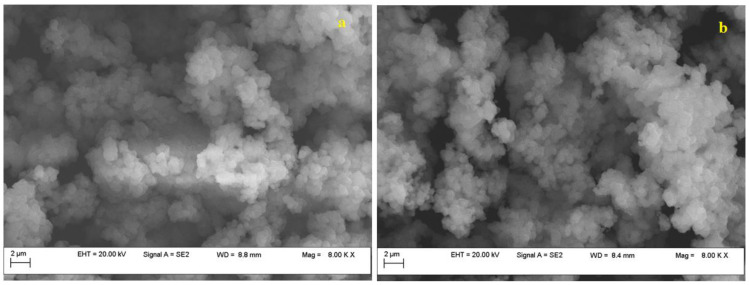
Fe-BTC SEM images, before (**a**) and after (**b**) adsorption experiments.

Both before and after the adsorption-desorption experiments, Fe-BTC was examined by scanning electron micrographs (SEM), and their analysis did not provide sufficient information to determine whether the adsorbate was physically present or whether the tested adsorbent underwent potential changes (Figure 4).

The Fe-BTC exhibited a homogeneous, dense structure, having granular-shaped nanoparticles with an average particle size of about 200 mm, consistent with other studies [39]. EDS analysis revealed the presence of peaks assigned to oxygen, carbon and Fe. The initial concentration of Fe was 12 wt.%, which, after adsorption, decreased to 7 wt.%, while oxygen increased from 49 wt.% up to 54 wt.%. Also, EDS confirmed the increase in C concentration, from 35 wt.% to 40 wt.%.

An investigation of the specific surface area and pore distribution was performed on the Fe-BTC nanomaterial (Figure S1—Supplementary Materials). The specific surface area of the sample was 360 m^2^/g, according to Brunauer–Emmett–Teller (BET), and the pore size distribution shows micropores with an average diameter of about 1.9 nm, as well as mesopores with an average diameter of around 2.4 nm. The N_2_ adsorption–desorption results showed that the sample has a porous structure at the boundary between micro- and mesopores, consistent with SEM morphology and literature data [40].

**Figure 5:**
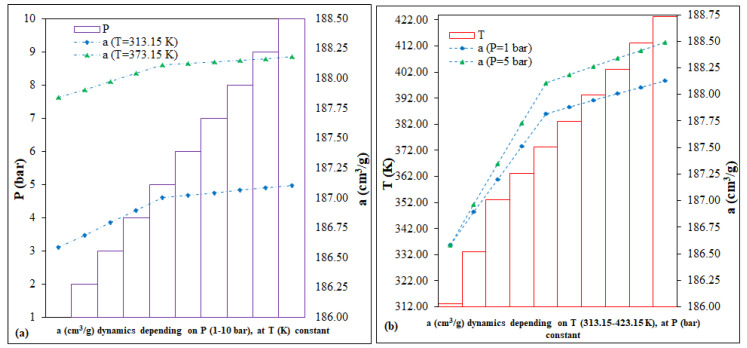
Fe-BTC predictions of adsorption capacity: (**a**) with variable P (bar) and constant T (K), (**b**) with variable T (K) and constant P (bar).

The calculation of predictions involves simulating scenarios by running a series of variables-drivers, coefficients and technical parameters, with the stated purpose of (i) saving resources, (ii) improvement and/or (iii) mitigation of hazards associated with the technological processes being implemented [41]. In the case of Fe-BTC, a range of variables were considered to predict the CO_2_ adsorption capacity. The key parameters, P (bar) and T (K), were selected based on the literature and third influence on the adsorption processes within the MOF column-reactor system, as depicted in Figure 5. The predictions of the adsorption capacity dynamics, *a* (cm^3^/g), were evaluated using the equations common to the linear interpolation and extrapolation functions [42]:

(4) $$y=\frac{y_{1}-y_{2}}{x_{2}-x_{1}}\times \left(x-x_{1}\right)+y_{1}$$

where y = independent variable; $x_{1}$ = first independent variable; $x_{2}$ = second independent variable; $y_{1}$ = function value at value $x_{1}$; $y_{2}$ = function value at $x_{2}$ value and

(5) $$y_{3}=y_{1}+\frac{x-x_{1}}{x_{2}-x_{1}}\times (y_{2}-y_{1})$$

where $x_{1-3}$ and $y_{1-3}$ are the coordinate points.

In accordance with the predictions (Figure 5), each of the following scenarios was represented by two fixed intervals: (i) the prediction as a function of P (bar), 1 and 5 vs. 1–10; (ii) prediction as a function of T (K), 313.15 and 373.15 vs. 313.15–423.15. In conclusion, for Fe-BTC, the two running variables, P (bar) and T(K), for 40 points, can have a low, different impact on the evolution of the dynamics of the CO_2_ adsorption capacity, as follows: (i) the P (bar) variation scenario, of <0.5%, for the interval 186.58–188.11 cm^3^/g; (ii) the variation scenario of T (K), of <0.5%, for the interval 186.58–188.49 cm^3^/g.

Recyclability is an important issue for solid adsorbents. Fe-BTC illustrates an adsorption capacity value over four adsorption–desorption cycles in the range of a_initial_ ± 10%.

### 3.2. Characterisation and Behaviour of UiO-66

The selective adsorption of CO_2_ in UiO-66 is based on the interactions that occur between the guest molecules and the topological or chemical characteristics of the MOF as a host. Although researchers have developed a rich library of UiO-66 that varies in shape, size and physicochemical functionality, a molecular-level understanding of how structure and functionality affect adsorption and transport has yet to be fully demonstrated.

UiO-66 involves octahedral nodes with a compact cubic unit cell structure. To date, the selective CO_2_ adsorption capacities for UiO-66, with incorporated functionalities, have been studied and ranged from 2.7 wt.% (T = 298 K) to 25.6 wt.% (T = 273 K) [43]. MOFs of the UiO-66 class are characterized by excellent chemical, thermal and mechanical properties [44]. Furthermore, UiO-66 is considered highly stable, with increased selectivity for CO_2_ adsorption, high workability and efficient recyclability [45]. The selectivity of this MOF indicates a higher adsorption capacity for CO_2_ compared to other gases, such as CH_4_, N_2_, CO and H_2_. The amount of CO_2_ adsorbed at T = 303 K, P = 0.99 bar can reach an adsorption capacity of 23.52 cm^3^/g [46]. The CO_2_ adsorption capacity value for UiO-66 was reported in the literature [46] to be 40.79 cm^3^/g (298 K, 1 bar), which is equivalent to the general adsorption capacity of UiO-66 (32.10 cm^3^/g). This measurement is comparable to the 54.22 cm^3^/g measurement made in this study (Table 3).

The selective adsorption capacity performance of UiO-66 was attributed to its high specific surface area, easily tuneable pore structure and high chemical and thermal stability. However, its CO_2_ adsorption amount is somewhat modest compared to that of Fe-BTC, which is in accordance with the literature [17].

The results of the experiments indicate two reasons for CO_2_ binding inside UiO-66: (i) the different vibrational frequencies of the O=C=O asymmetric stretching mode, a configuration made by -H bonds with μ_3_-OH groups on the UIO-66 nodes and (ii) dispersive interactions, where the topological features of the MOF affect the adsorption of GHG gas molecules [47].

The smaller pore diameters in UiO-66 were principally responsible for the selective CO_2_ absorption; nevertheless, the presence of N_2_ atoms in the pores appears to be advantageous for improving selectivity and mechanically aids in channel narrowing [48]. Knowing that UiO-66 contains two types of pores—octahedral and tetrahedral—which differ mostly in the orientation of the terephthalic bonds so that, in the tetrahedral ones, the organic bonds are closer to the interior of the pores by their size (tetrahedral (8 Å) and octahedral (11 Å)), the working pressure of the experiments (P = 1–5 bar) favoured more than 95% filling of the tetrahedral pores with CO_2_, the adsorption in the octahedral pores being possible at higher pressures. This observation may lead to the conclusion that the CO_2_ molecules fit perfectly in the corners of the tetrahedral pores, but not in the octahedral pores. This is attributed to the known size entropy effect of CO_2_ in the pores [48]. In other words, tetrahedral pores contain adsorption zones with affinity for CO_2_ and octahedral pores do not [49]. Validation of the selective adsorption capacity of UIO-66 was investigated using gas chromatography, EA and FTIR.

**Figure 6:**
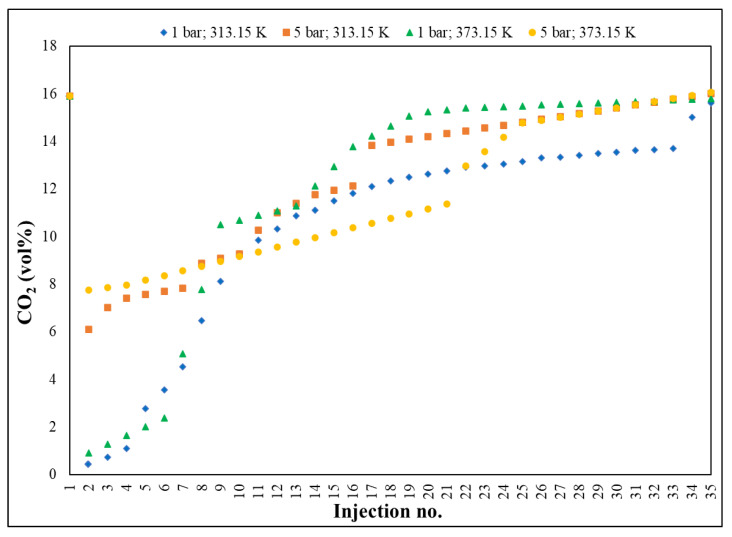
UIO-66—evolution of selective CO_2_ adsorption by GC investigation.

Gas chromatography (Figure 6) investigated the evolution of the CO_2_ concentration in a gaseous mixture on three segments: the first, between 15.89–0.44 vol%, with a decrease rate of 10.92 vol%; the second, 0.44–10.31 vol%, characterized by an adsorption plateau, with a rate of 3.78 vol% and the third, 10.31–15.62 vol%, representing the saturation period, with an increase rate of 1.20 vol%, with an average rate of 0.64 vol%, from one injection to another of the sample.

Elemental analysis (EA) involved the determination of C (wt.%) concentrations in the UiO-66 samples, and the results revealed an increase in the C (wt.%) content at the end of the experiment in relation to the initial levels: C_initial_ = 31.00 wt.% vs. C_final_ = 35.34 wt.%.

**Figure 7:**
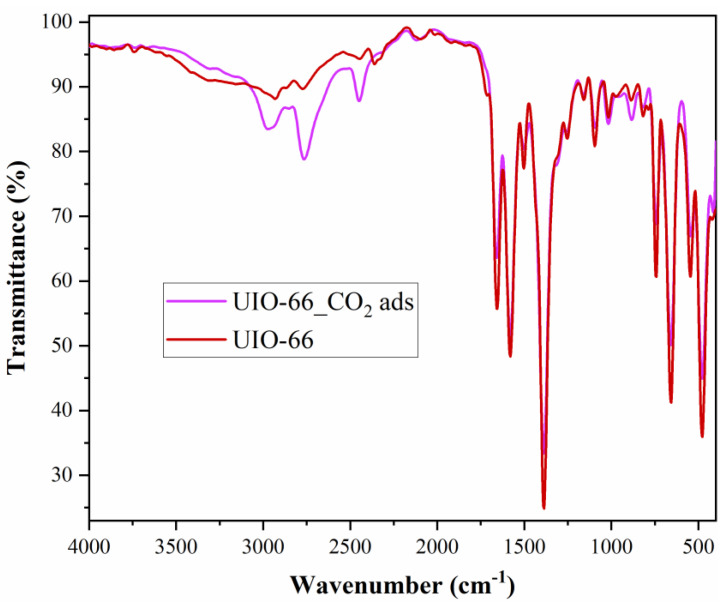
UiO-66 FTIR spectra before and after adsorption.

As in the case of Fe-BTC, FTIR analysis (Figure 7) confirmed the presence of CO_2_ molecules on the surface of the nanomaterial.

The spectra of both samples are consistent with the literature and show intense bands in the 1700–1250 cm^−1^ region, due to the carboxylate groups and deformations of the phenyl ring [50]. The bands in the region 3100–2400 cm^−1^ are due to C-H stretching modes. A sharp and narrow bit is detected at 3742 cm^−1^ and is attributed to free hydroxyls—μ3 belonging to Zr6 groups [50]. The bands at 1580 and 1390 cm^−1^ were associated with esters and residual terephthalic acid [50]. The band at 1658 cm^−1^, together with a corresponding weak band at 1092 cm^−1^, originates from residual DMF. The intense CO_2_ ν_3_ (O=C=O asymmetric stretching) mode registered at about 2500 cm^−1^ and the weaker CO_2_ combination bands (ν_3_ + 2ν_2_ and ν_3_ + ν_1_) from 2700 and 2950 cm^−1^ confirmed the existence of CO_2_ in the sample [51]. The literature confirmed that the CO_2_ combination modes can be applied for monitoring the CO_2_ insertion into the nanoporous materials pores, the CO_2_ behaviour, as well as the matrix–guest interaction [51].

**Figure 8:**
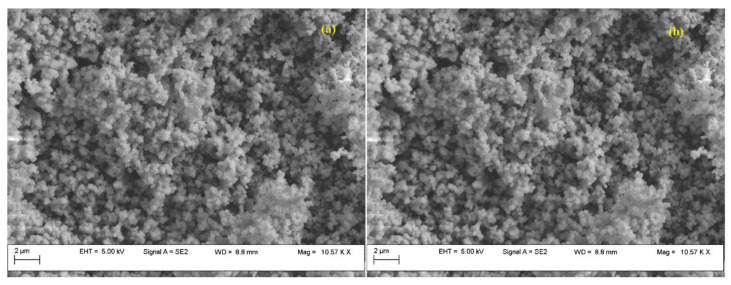
UiO-66 SEM images before (**a**) and after (**b**) the adsorption.

SEM analysis of the UiO-66 sample (Figure 8), before and after the adsorption–desorption experiments, was not conclusive in evaluating the CO_2_ presence.

Both before and after adsorption, UiO-66 presents a homogeneous dense structure. In can be observed that UiO-66 consists of nano-sized crystals of octahedral shape. UiO-66 exhibits a narrow crystal size distribution between 150 and 250 nm [50]. Instead, EDS analysis revealed an increase in C concentration (from 31 wt.% up to 35 wt.%), confirming the hypothesis that CO_2_ was adsorbed on the MOF surface. Also, EDS confirmed the presence of the Zr metal at a concentration of 41 wt.%, which decreased after adsorption to 40%; the oxygen concentration increased from 18 wt% to 21%, also confirming the CO_2_ presence.

The UiO-66 material was also characterized by specific surface area and pore distribution (Figure S2—Supplementary Materials). The specific surface area of the sample was 811 m^2^/g, according to Brunauer–Emmett–Teller (BET), and the pore size distribution shows micropores with an average diameter of about 1.5–1.7 nm, as well as mesopores with diameter of 2.9 nm. The N_2_ adsorption–desorption results showed that the sample has a porous structure at the boundary between micro- and mesopores, consistent with SEM morphology and literature data [52].

**Figure 9:**
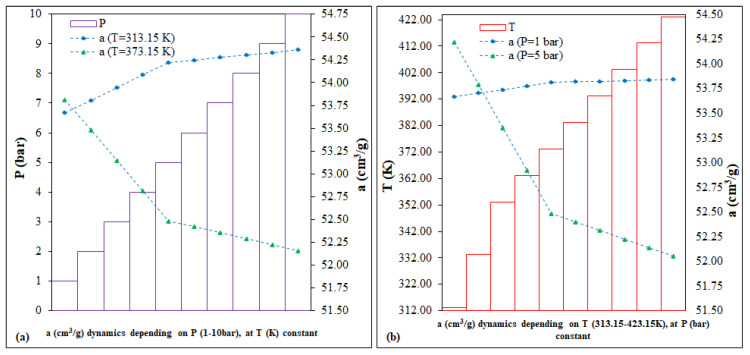
UIO-66. Predictions of adsorption capacity: (**a**) with variable P (bar) and constant T (K), (**b**) with variable T (K) and constant P (bar).

The scenarios for UiO-66 ran two variables, P (bar) and T (K), with different impacts on the dynamic evolution of the CO_2_ adsorption capacity, as follows: (i) scenario of P (bar) variation, by ~1.5%, for the interval 52.16–54.36 cm^3^/g and (ii) the T (K) variation scenario, by ~1.3%, for the interval 52.05–54.22 cm^3^/g (Figure 9).

### 3.3. Characterization and Behaviour of the Cu–Zn–MCM-41

Mesoporous MCM-41 type materials are considered potential candidates for efficient adsorbents, due to their structure that facilitates the flow of GHGs [53]. The literature presents MCM-41 functionalized with monoethanolamine with a maximum CO_2_ adsorption capacity of 27.78–39.26 cm^3^/g at T = 298.15 K and P = 1 bar [54]. Also, MCM-41 adsorbent was modified with monoethanolamine and diethanolamine to improve CO_2_ adsorption, registering high CO_2_ adsorption capacity, even after 10 operating cycles [55]. The synthesized nanomaterial, denoted as Cu–Zn–MCM-41, proved further selectivity towards CO_2_ adsorption due to its physicochemical properties, which is in accordance with the literature [56]. The explanation regarding the selective adsorption capacity can be related to the 2D hexagonal structure and the adjustable pore diameter. Fast adsorption kinetics and maximum recorded adsorption capacity of 33.95 cm^3^/g CO_2_ were demonstrated using the developed column-reactor system (Table 4). The homogeneous arrangement in the reactor column of the filler (Cu–Zn–MCM-41) around the quartz balls—between the metal sandwich screens—allowed the contact surface to increase and the reaction time between the adsorbent and the adsorbate to be high [57,58]. The study of the physical adsorption mechanism of CO_2_ suggested capillary condensation caused by the nanoscale channels of Cu–Zn–MCM-41 as the main contribution [59].

**Figure 10:**
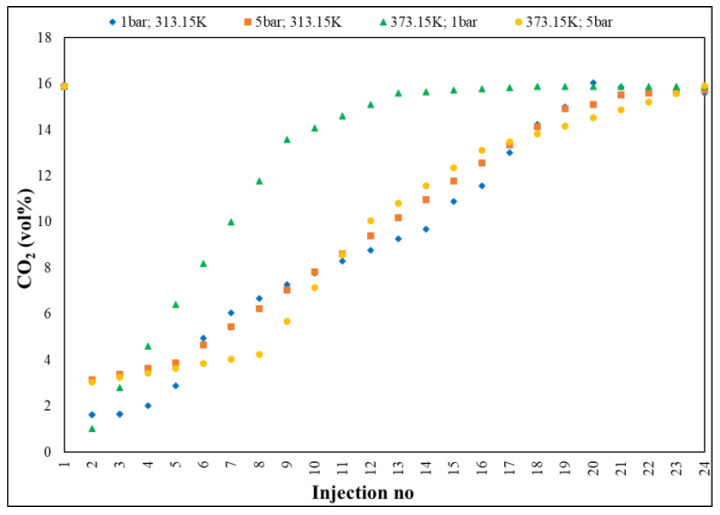
Cu–Zn–MCM-41: evolution of selective CO_2_ adsorption by GC investigation.

Figure 10 presents the CO_2_ adsorption at T = 313.15–373.15 K, P = 1–5 bar and Q = 100 mL/min, where, in independent stages, (i) pure N_2_ passed through the silica surface in the first seconds, giving the background absorbance; (ii) CO_2_ replaced N_2_ in the flow and it was adsorbed on the silica surface; (iii) the absorbance corresponding to the process increases rapidly to reach an equilibrium state. The physical adsorption and rate were significantly improved with increasing pore size, a behaviour in contrast to that exhibited by Fe-BTC and UIO-66, due to the order degree, i.e., high crystallinity, this being consistent with a potential for higher interaction between the adsorbate and the adsorbent. Also, the weak van der Waals forces had a contribution to the physical adsorption capacity, an exothermic one—the increase in the isosteric heat between the adsorbent and adsorbate interface [20,60]. The cyclic stability of the developed Cu–Zn–MCM-41 was evaluated using reproducible adsorption–desorption experiments—under the same technical conditions—and the characterization results showed that the target adsorbents reacted successfully, showing good renewability and stability over the predetermined thermal range, 313.15–373.15 K on adsorption, at 313.15–298.15 K on desorption, with RSD ± 10%, at the end of 4 cycles [61,62]. The conclusive results place Cu–Zn–MCM-41 in the class of promising CO_2_ adsorbents.

Elemental analysis (EA) revealed an increase in the concentration of C (wt.%) at the end of the experiment in relation to the initial level, from C_initial_ = 0.79 wt.% to C_final_ = 3.04 wt.%.

**Figure 11:**
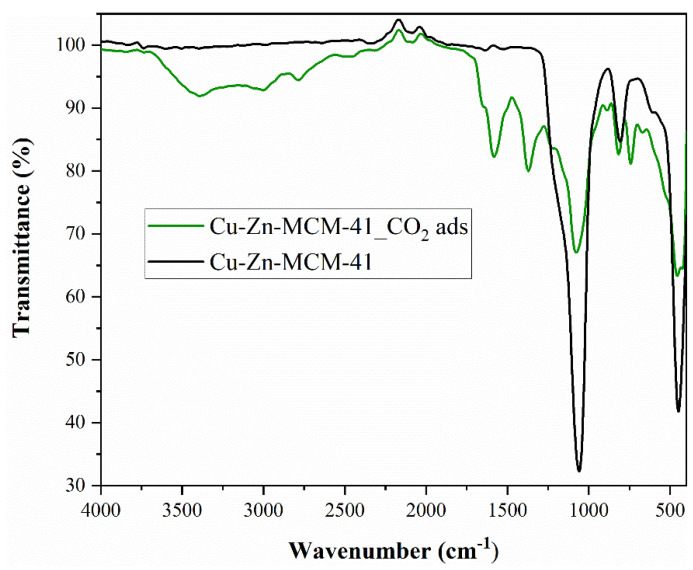
Cu–Zn–MCM-41 FTIR spectra before and after adsorption.

As in the case of both MOFs, FTIR analysis (Figure 11) confirmed the presence of CO_2_ molecules on the surface of the nanomaterial.

The peak from 1632 cm^−1^ corresponds to the deformation mode for the surface hydroxyl groups. The two bands from 1051 cm^−1^ and 805 cm^−1^ were assigned to stretching vibrations for symmetric and asymmetric Si-O clusters. The strain vibrations for the Si-O- groups on the silica surface were highlighted by the appearance of one peak at about 447 cm^−1^ [63]. Metallic interactions lead to increased distortions and vibrations of the silicon–oxygen tetrahedron, resulting in a more pronounced asymmetry [56]. When the metals were introduced, the FTIR spectrum suffered some changes: a shoulder was registered at 577 cm^−1^, attributed to Si-O-metal vibrations, concluding that metallic species were successfully impregnated on MCM-41. The introduction of metals was also confirmed by the disappearance of the peak at 615 cm^−1^ specific to stretching vibrations for the Si-O- groups on the silica surface, confirming the positioning of the metal on the silica surface. When CO_2_ was adsorbed on Cu–Zn–MCM-41, the peaks specific to the silica support decreased in intensity, demonstrating that CO_2_ targeted the silanol surface (not the areas where the metallic species were bonded). Also, the band and peaks characteristic of CO_2_ appeared.

The presence of prominent peaks at 1375 cm^−1^ and 1580 cm^−1^ may indicate the formation of carbamic acid, the first one being attributed to δ_OH_, and the second one to C=O vibration [64]. One can observe a large band at about 3400 cm^−1^ which can be attributed to physically adsorbed water, probably present in the gas, adsorbed during the experiments or to -OH stretching of free surface OH groups [64]. The peaks from 2780–2990 cm^−1^ were attributed to C-H binding, which can be explained by the bond between the OH groups from the silica surface and the C from CO_2_ [64].

The FTIR analysis indicated that CO_2_ not only is physically adsorbed on the silica surface, but most likely is also trapped as carbamic acid or carbamate species, corresponding to previously conducted studies regarding CO_2_ binding on silica materials [65].

**Figure 12:**
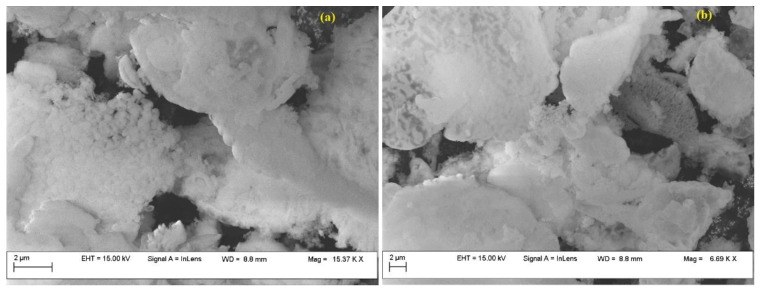
Cu–Zn–MCM-41 SEM images before (**a**) and after (**b**) the adsorption.

As in the case of both MOFs, SEM analysis was inconclusive in revealing the presence of CO_2_ at the nanomaterial surface (Figure 12).

Scanning electron microscopy revealed the morphology of the sample (Figure 12). The bimetallic system presented spherical and partially elongated particles with dimensions below 100 nm, the ordered morphology typical of MCM-41 mesoporous silica being maintained. EDS analysis confirmed Cu and Zn content. EDS also confirmed that, after CO_2_ adsorption, the C and O percent increased, from 0.8 wt.% up to 3 wt.% and from 46 wt.% up to 49 wt.%.

The Cu–Zn–MCM-41 nanomaterial was also characterized by specific surface area and pore distribution (Figure S3—Supplementary Materials). The specific surface area of the sample was 607 m^2^/g, according to Brunauer–Emmett–Teller (BET), and the pore size distribution shows mesopores with diameter of 3.5 nm. The mesopore volume decreased drastically when metals were introduced into the silica matrix, down to 0.492 cm^3^/g, one possible explanation being that the metals have entered the mesoporous silica matrix [56].

**Figure 13:**
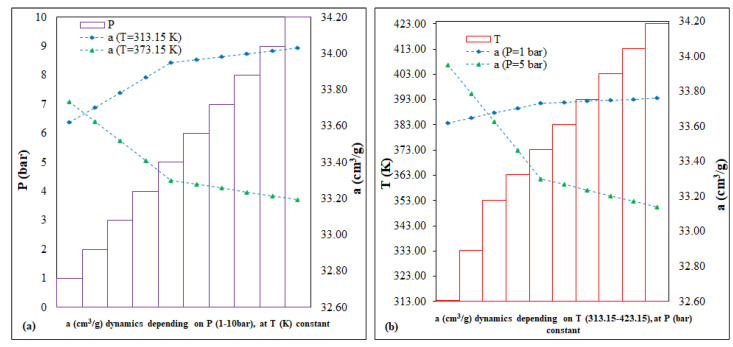
Cu–Zn–MCM-41—predictions of adsorption capacity: (**a**) variable P (bar) and constant T (K), (**b**) variable T (K) and constant P (bar).

For Cu–Zn–MCM-41, the two run variables had a direct impact on the evolution of the CO_2_ adsorption capacity dynamics: (i) the P (bar) variation scenario, of <1%, for the interval 33.19–34.03 cm^3^/g; (ii) the T (K) variation scenario, of <1%, for the interval 33.14–33.95 cm^3^/g (Figure 13).

## 4. Discussion

**Figure 14:**
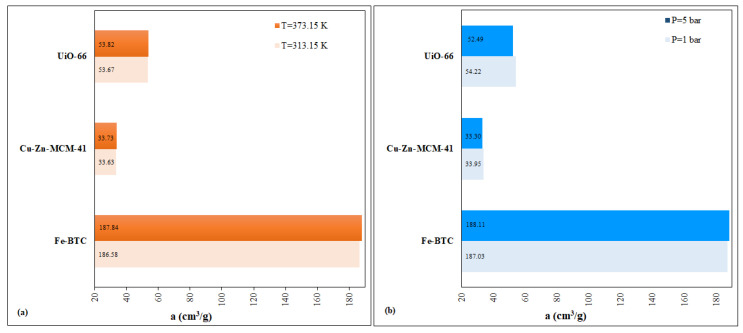
Nanomaterials’ adsorption capacity—temperature variation (**a**), pressure variation (**b**).

The tests developed in the present study, run in a column-reactor system, required a comparative evaluation regarding the performances of the selected nanomaterials Fe-BTC, UIO-66 and Cu–Zn–MCM-41. The evaluation was achieved in the following conditions: (i) the gas subjected to the adsorption test—CO_2_; (ii) T = 313.15–373.15 K; (iii) P = 1–5 bar (Figure 14).

The study demonstrated that the developed MOFs recorded a higher CO_2_ adsorption capacity with increase in temperature: Fe-BTC > UIO-66 > Cu–Zn–MCM-41 (Figure 14a). In the pressure case, the increase/decrease in the absorption capacity does not follow a pattern, each material having its own specificity (sinusoidal type) without the possibility to extrapolate the situation (Figure 14b). Regarding the average values, temperature was the most influential variable in increasing CO_2_ adsorption, whereas pressure proved decisive for one of the MOFs, i.e., for Fe-BTC.

The predictions regarding the adsorption capacity were evaluated through four types of scenarios and regression analysis was applied for each of them, in order to estimate (i) the relationship between the quantitative variables/the strength of the relationship, (ii) the value of the dependent variable at a certain value of the independent variable and (iii) model fitting. The dependent variable is a (cm^3^/g) and the independent variables were T = 313.15–423.15 K and P = 1–10 bar. Multiple R, i.e., the Pearson coefficient (Table 5), varying from −1 to +1, was used to evaluate the strength of the linear relationship between the predictor variables and the response variable [66]. It was between the values of 0.92 and 0.95, which indicates a strong correlation relationship between the predictors, P and T, and the response variable, a (cm^3^/g).

Coefficient of determination, r^2^, is the proportion of variance in the response variable that can be explained by the predictor variables [67]. The value of r^2^ (0.87–0.90)—Table 6—indicates that >87% of the variance in adsorption capacity levels obtained can be explained by the pressures and temperatures set for the column-reactor system.

The *p*-value (0.0001–0.0003—Table 7) was less than the common significance level of 0.05. This indicates that the regression model is statistically significant (the model fits the data better than the model with no predictor variables) [68]. The statistical analysis using *p* < 0.05 indicates that there are significant changes in adsorption capacity values with changes in technological parameters [69].

## 5. Conclusions

Considering the physicochemical methods used in the present study as standard analytical tools for assessing the viability of CO_2_ adsorption operations, it is necessary to critically analyse the results obtained for each of the evaluated MOFs under similar conditions of pressure, temperature and flow in the same column-reactor system. Several key observations can be made: (i) the unique design and the unitary character of the installation, along with the arrangement of the active filling inside the reactor column, allowed an increase in the contact surface area between adsorbate and adsorbent, favouring the physical adsorption process for each tested nanomaterial; (ii) the highest dynamic of selective adsorption capacity was recorded for Fe-BTC, a: 188.11 cm^3^/g CO_2_; (iii) both temperature and pressure proved to be determining factors in increasing the selective adsorption capacity. Moreover, we show that this hybrid approach—in situ experiments (which are based on the existence of a pressure and temperature domain) and scenarios/forecasts—is a modern/efficient way to accurately predict the adsorption behaviour of developed solid materials. These findings provide valuable insights for the further optimization of the already reported conditions for the adsorption and storage of CO_2_, released into the atmosphere by electric and thermal power plants, using nanomaterials, replacing the classic amines, through a system like the one developed in this study. The experimental processes highlight an encouraging dynamic of the CO_2_-selective adsorption capacity, aligning with the results reported in the specialized literature. This opens possibilities for advancement to a higher level, such as the design of a pilot facility for the capture and storage of GHGs.

## Figures and Tables

**Table 1:** CO_2_ levels in the flue gases (vol%).

Fuel Type	Sample IDs				
Sampling month	November	December	January	February	March
Number of samplings/month	32				
CO_2_ Average/Coal (lignite)	15.020	15.020	15.020	15.020	15.020
CO_2_ Average/Natural gas	4.114	3.047	3.956	3.177	4.008

**Table 2:** Fe-BTC column-reactor system (*CRS*) experiment sheet.

Technological Parameters (Variables)			Validation of the CRS		
T_reactor column_ (K)	P (bar)	t_breakthrough time, min_ (CO_2_)	a (cm^3^/g)	η, (%)	R (%*v.v*)
313.15	1	40.00	186.58	99.87	97.43
313.15	5	8.64	187.03	92.70	88.97
373.15	1	40.32	187.84	99.75	94.03
373.15	5	8.46	188.11	95.22	92.69
V_reactor column_ (cm^3^)	510				
Q_test gas mixture_ (mL/min)	100				
m (g)	~4.5				
m_quartz balls_ (g)	~335				

**Table 3:** UiO-66 column-reactor system experiment sheet.

Technological Parameters (Variables)			Validation of the CRS		
T_reactor column_ (K)	P (bar)	t_breakthrough time, min_ (CO_2_)	a (cm^3^/g)	η, (%)	R (vol%)
313.15	1	12.00	53.67	97.22	97.22
313.15	5	3.83	54.22	61.55	61.55
373.15	1	12.41	53.82	94.27	94.27
373.15	5	4.46	52.49	51.16	51.16
V_reactor column_ (cm^3^)	510				
Q_test gas mixture_ (mL/min)	100				
m (g)	~8.2				
m_quartz balls_ (g)	~337				

**Table 4:** Cu–Zn–MCM-41 column-reactor system experiment sheet.

Technological Parameters (Variables)			Validation of the CRS		
T_reactor column_ (K)	P_working_ (bar)	t_breakthrough time, min_ (CO_2_)	a, cm^3^/g	η, (%)	R (vol%)
313.15	1	8.00	33.63	89.74	89.79
313.15	5	1.81	33.95	78.67	79.42
373.15	1	7.70	33.73	82.32	87.97
373.15	5	1.76	33.30	79.61	80.24
V_reactor column_ (cm^3^)	510				
Q_test gas mixture_ (mL/min)	100				
m (g)	~1.2				
m_quartz balls_ (g)	~340				

**Table 5:** Pearson’s correlation matrix for all the features included in the study predictive performance.

Regression			
Technological Parameters (as Dataset) P = 1–10 bar			
T (K)	Fe-BTC	UiO-66	Cu–Zn–MCM-41
313.15	0.9272	0.9270	0.9271
373.15	0.9272	0.9271	0.9272
313.15–423.15	0.9477	0.9476	0.9477
313.15–423.15	0.9476	0.9477	0.9476

**Table 6:** Coefficient of determination matrix for all features included in the study predictive performance.

Regression			
Technological Parameters (as Dataset) P = 1–10 bar			
T (K)	Fe-BTC	UiO-66	Cu–Zn–MCM-41
313.15	0.8596	0.8596	0.8594
373.15	0.8597	0.8595	0.8596
313.15–423.15	0.8981	0.8981	0.8981
313.15–423.15	0.8980	0.8982	0.8981

**Table 7:** *p* statistic matrix for all the features included in the study predictive performance.

Regression			
Technological Parameters (as Dataset) P = 1–10 bar			
T (K)	Fe-BTC	UiO-66	Cu–Zn–MCM-41
313.15	0.0003	0.0004	0.0003
373.15	0.0003	0.0002	0.0002
313.15–423.15	0.0001	0.0001	0.0001
313.15–423.15	0.0001	0.0001	0.0001

## Data Availability

The data presented in this study are available on request from the corresponding authors. The data are not publicly available due to institutional policies.

## Supplementary Materials

The supporting information can be downloaded at: https://www.mdpi.com/article/10.3390/nano13192637/s1.
