# Supplementary material for: Selectivity of MOFs and Silica Nanoparticles in CO2 Capture from Flue Gases
Source: Nanomaterials (Basel). 2023 Sep 25;13(19):2637. doi: 10.3390/nano13192637 (PMC10574321; doi:10.3390/nano13192637)
Supplement: Supplementary file 1 [file nanomaterials-13-02637-s001.zip › nanomaterials-2610899-supplementary.pdf]

- *Supplementary material -*

Testing the nanomaterials activity and selectivity in reactions for CO_2 reduction from flue gases

Felicia Bucura[1], Stefan-Ionut Spiridon[1], Roxana Elena Ionete[1], Florian Marin[1], Anca Maria Zaharioiu[1], Adrian Armeanu[1], Silviu-Laurentiu Badea[1], Oana Romina Botoran[1], Eusebiu Ilarian Ionete[1], Violeta-Carolina Niculescu[1,*], Marius Constantinescu[1,*]

[1]National Research and Development Institute for Cryogenic and Isotopic Technologies - ICSI Ramnicu Valcea, 4 Uzinei Street, P.O. Box Raureni 7, 240050, Rm. Valcea, Romania

* Correspondence: violeta.niculescu@icsi.ro; marius.constantinescu@icsi.ro

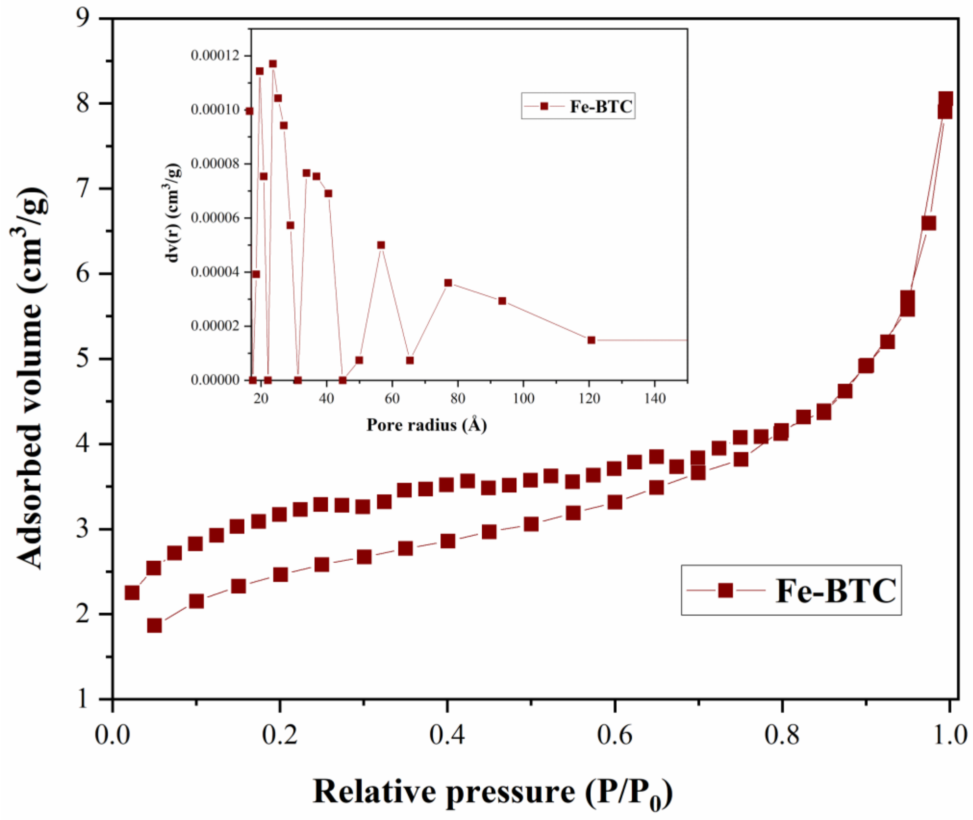

Figure S1. Fe-BTC. Specific surface area and pore distribution

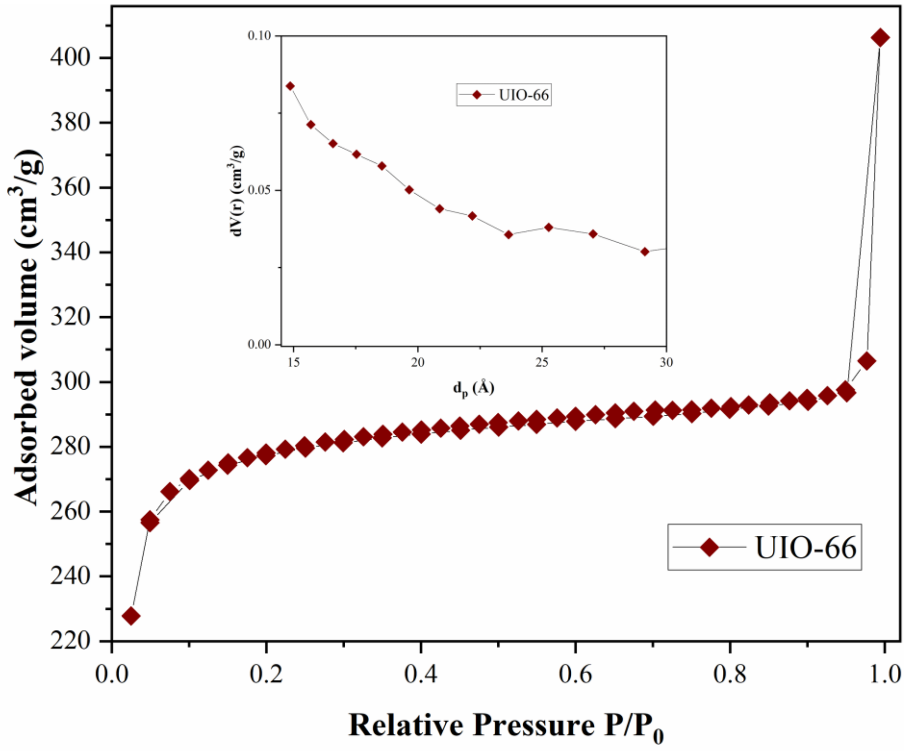

Figure S2. UiO-66. Specific surface area and pore distribution

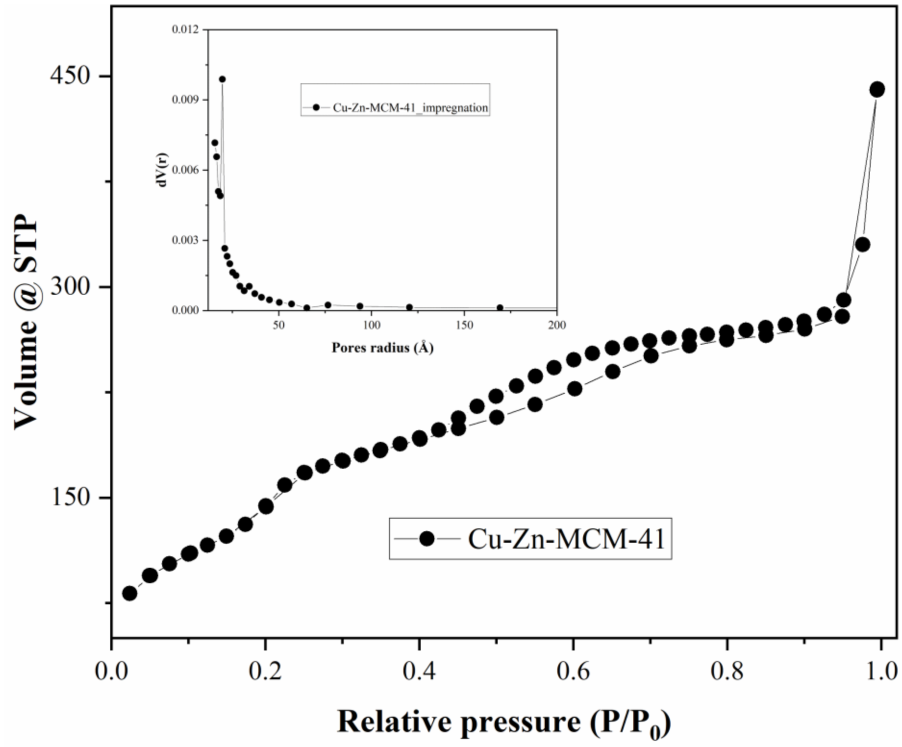

Figure S3. Cu-Zn-MCM-41. Specific surface area and pore distribution
